# Phantom simulation of liver metastasis on a positron emission tomography with computed tomography scan after neoadjuvant chemoradiotherapy for distal esophageal cancer: a case report

**DOI:** 10.1186/s13256-020-02391-z

**Published:** 2020-07-07

**Authors:** Sen-Ei Shai, Yuan-Hong Lin, Yi-Ling Lai, Hsiao-Wen Tang, Yao-Wen Hsieh, Shih-Chieh Hung

**Affiliations:** 1 Division of Thoracic Surgery, Taichung Veterans General Hospital, 1650 Taiwan Boulevard Sect. 4, Taichung, Taiwan; 2 Institute of Clinical Medicine, National Yang-Ming University, Taipei, Taiwan; 3 Department of Pathology, Veterans General Hospital, 1650 Taiwan Boulevard Sect. 4, Taichung, Taiwan; 4 National Chi Nan University, Nantou, Taiwan; 5 Institute of New Drug Development, China Medical University, Taichung Joint PI, IBMS, Academia Sinica 7F, No. 6, Xueshi Road, North District, Taichung City, 404 Taiwan; 6 Integrative Stem Cell Center, China Medical University Hospital, Taichung Joint PI, IBMS, Academia Sinica 7F, No. 6, Xueshi Road, North District, Taichung City, 404 Taiwan

**Keywords:** Esophageal cancer, Radiation therapy, PET-CT scan, Liver metastasis

## Abstract

**Background:**

Neoadjuvant chemoradiotherapy is currently the gold standard treatment for esophageal cancer prior to surgery. This radiation therapy will sometimes lead to liver damage parallel to esophageal lesions, which mimics liver metastasis visualized by ^18^F-fluorodeoxyglucose positron emission tomography with computed tomography. In this report, we publish virtual radiation-induced liver damage images obtained during surgery, along with the coherent pathology, in order to confirm the false-positive result through an optimally decisive radiological examination.

**Case presentation:**

We report a case of a Asian male patient with distal esophageal cancer who had undergone neoadjuvant chemoradiotherapy (5000 cGy). Subsequently, a new lesion was discovered during a positron emission tomography with computed tomography scan 6 weeks later, near the left caudate lobe of the liver during tumor restaging. To exclude the possibility of liver metastasis, serial imaging was conducted, which included liver sonography, computed tomography, and magnetic resonance imaging for a more intimate probe. The patient’s condition was verified as being liver inflammation change, as seen by the liver magnetic resonance imaging presentation. Thoracoscopic esophagectomy was performed with cervical esophagogastrostomy via the retrosternal route, along with a feeding jejunostomy. The procedure was performed smoothly, with an intraoperative liver biopsy also being conducted 2 weeks later, after positron emission tomography with computed tomography restaging. The pathology report revealed esophageal cancer in the form of poorly differentiated squamous cell carcinoma, pT3N1M0. The liver biopsy revealed obvious inflammation change after radiation therapy, which elucidated sinusoidal congestion with the attenuated hepatic cords and filled with erythrocytes. There was no evidence of liver metastasis. The patient recovered uneventfully and was discharged with his oral intake performing smoothly, and a stable condition was observed during 12 months of outpatient department follow-up.

**Conclusions:**

New foci of increased ^18^F-fluorodeoxyglucose avidity are commonly seen in the caudate and left hepatic lobes of the liver during neoadjuvant chemoradiation for distal esophageal cancer, and these findings generally reflect radiation-induced liver disease rather than metastatic disease. Awareness of the pitfalls of a high ^18^F-fluorodeoxyglucose uptake in radiation-induced liver injury is crucial in order to avoid misinterpretation and overstaging. Except for the location of ^18^F-fluorodeoxyglucose uptake, the shape of the lesion, and an maximum standardized uptake value (> 10/h), a convincing liver magnetic resonance imaging scan or even a liver biopsy can provide accurate information for distinguishing radiotherapy-induced liver injury from liver metastasis.

## Introduction

Esophageal cancer is currently the eighth most common cancer worldwide. It is most prevalent in countries in eastern and southern Africa and eastern Asia. Three-fourths of affected patients are male. Patients diagnosed with esophageal cancer have a very poor survival rate [1]. There is evidence that neoadjuvant chemoradiotherapy (CRT) will shrink tumor size, which increases the success rate of surgery. Additionally, it can improve the average 3-year survival rate, with the radiation reducing locoregional cancer recurrence [2]. Positron emission tomography with computed tomography (PET-CT) scans are currently performed as the gold standard approach for tumor staging of esophageal cancer worldwide [3, 4]. The scans can be helpful in identifying the subset of patients who are responders to preoperative therapy while also detecting distant metastases, thereby excluding those patients who will not benefit from an esophagectomy [5]. Radiation therapy (RT) of distal esophageal cancer may include the adjacent liver parenchyma, which can appear on a PET-CT scan as an area of increased ^18^F-fluorodeoxyglucose (FDG) uptake within the liver parenchyma that mimics metastatic disease [5]. Approximately 3–10% of patients are detected with new lesion uptake after the procedure, which may be attributed to interval metastasis [4, 6].

Use of PET-CT for restaging may encounter a dilemma in distinguishing radiation-induced hepatitis from the tumor with liver metastasis, which in turn may accentuate the false-positive rate of PET-CT with overstaging [7, 8]. Accordingly, in this report, we describe a case with complete images from both pre- and post-neoadjuvant CRT, as well as a photograph of a damaged liver taken during surgery, along with a pathologic image. We also review the relevant literature regarding optimal detection to assist in excluding PET-CT false-positive misinterpretation after neoadjuvant chemoradiation.

## Case presentation

**Fig. 1:**
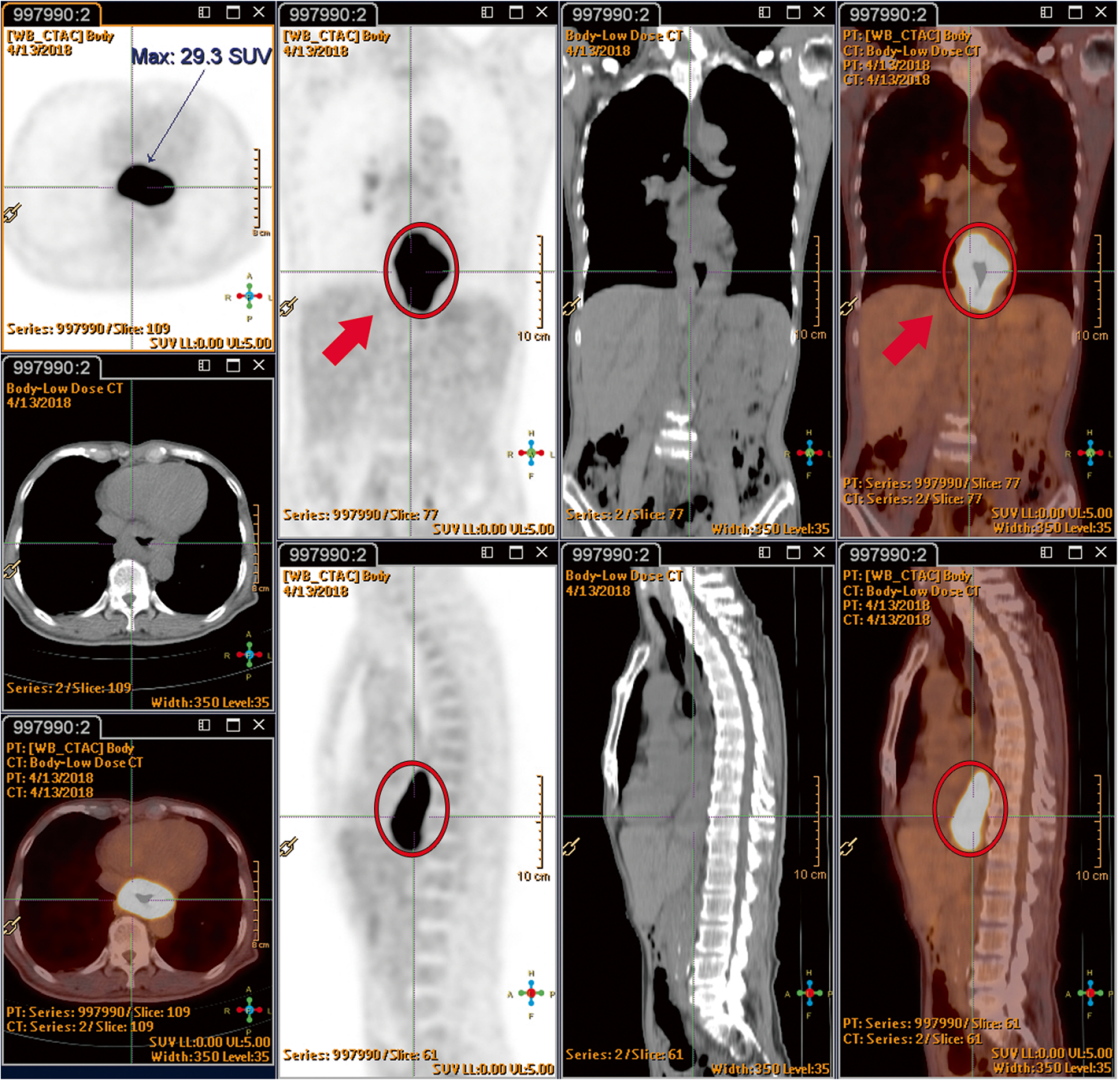
Prior to neoadjuvant chemoradiotherapy, the esophageal tumor shows high ^18^F-fluorodeoxyglucose uptake (9.7 × 5.6 cm, maximum standardized uptake value 29.3/1 hour) (*red circles*). Prior to neoadjuvant chemoradiotherapy, there are no active lesions seen in liver segment I (*red arrows*)

**Fig. 2:**
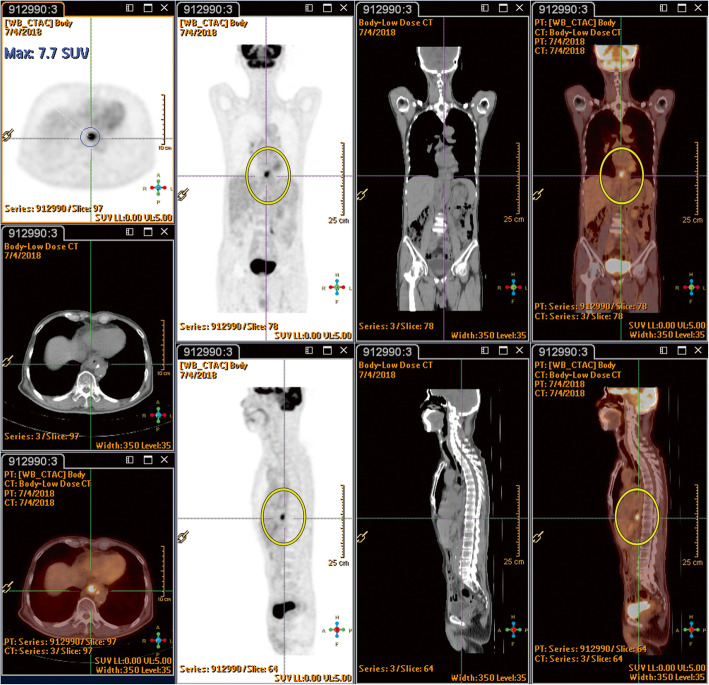
Six weeks after neoadjuvant chemoradiotherapy, the esophageal tumor had regressed (2.1 × 1.6 cm, maximum standardized uptake value 7.7/1 hour; indicated by *yellow circles*)

**Fig. 3:**
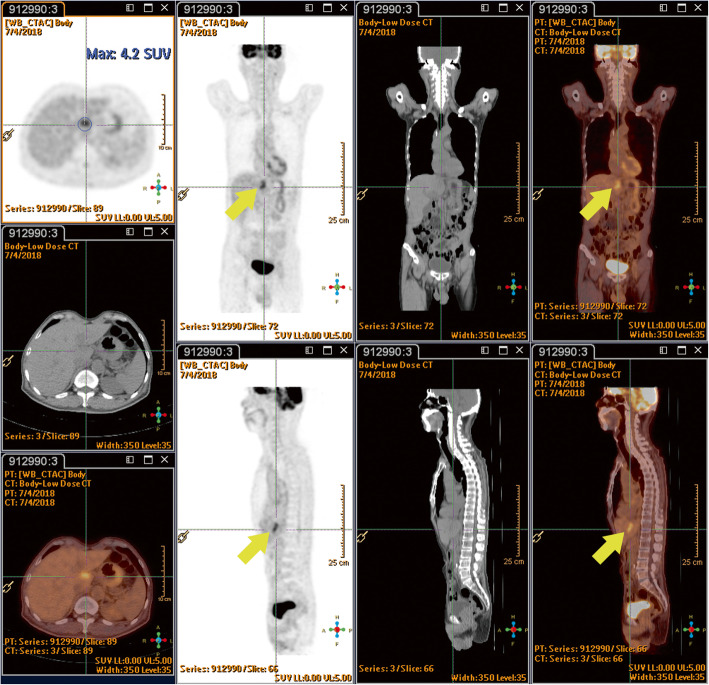
Six weeks after neoadjuvant chemoradiotherapy, a new ^18^F-fluorodeoxyglucose-avid lesion at liver segment I (3.5 × 1.5 cm, maximum standardized uptake value 4.2/1 hour; indicated by *yellow arrows*)

A 66-year-old Asian man who had hypertension for which he had been receiving medication for 20 years presented to our hospital. He had been a heavy smoker and drinker for more than 30 years. He had experienced progressive swallowing difficulty for 3 months and a body weight loss of 5 kg in 1 month. Endoscopy disclosed that a huge tumor was occupying the esophagus 30 cm from the incisor to the esophagogastric junction, with a biopsy determining poorly differentiated squamous cell carcinoma. A whole-body PET-CT scan with ^18^F-FDG revealed a distinct FDG uptake in the lower esophagus (Fig. 1), and clinical stage cT3N1M0 was subsequently categorized. A chemoport was inserted, and a percutaneous endoscopic gastrostomy was performed prior to neoadjuvant CRT. The patient underwent a two-cycle chemotherapy regimen with cisplatin and 5-fluorouracil, in combination with RT in 25 fractions at 5000 cGy, with no obvious side effects being noted. Following the feasibility of definitive surgery for esophageal resection and reconstruction, a PET-CT scan was performed again 6 weeks later upon completion of CRT in order to evaluate the effects of the neoadjuvant CRT (Fig. 2). It was at this time when a new FDG-avid lesion at the caudate lobe was discovered, and a formal report was written to address the liver metastasis (Fig. 3).

**Fig. 4:**
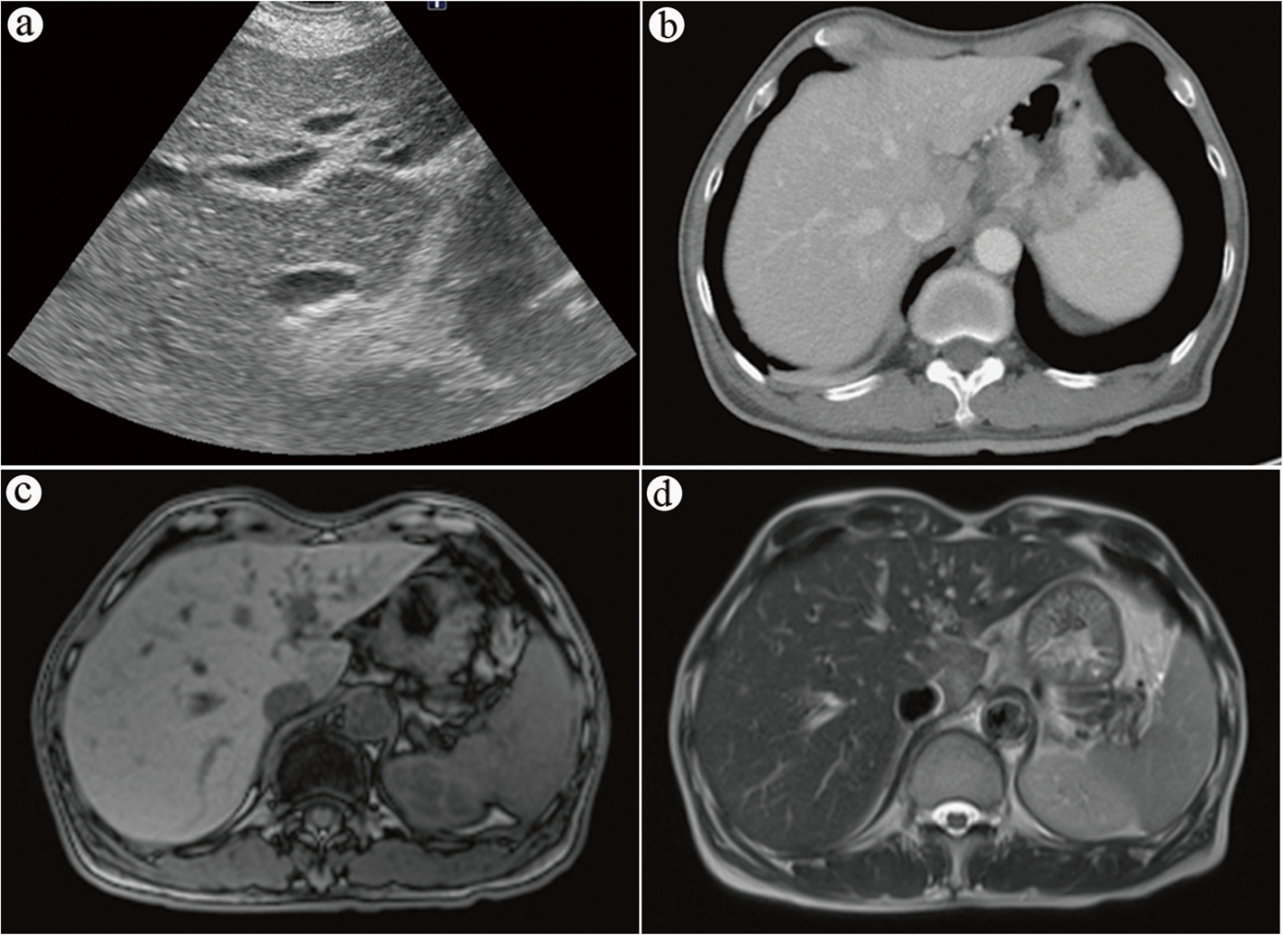
**a** Sonography of the liver shows hypoechoic appearance over caudate lobe. **b** Contrast-enhanced computed tomography shows focally less enhancement in S1 of the liver. **c** Magnetic resonance imaging (MRI) of the liver shows a low signal intensity over the caudate lobe in T1-weighted images. **d** MRI of the liver shows a high signal intensity over the caudate lobe in T2-weighted images

**Fig. 5:**
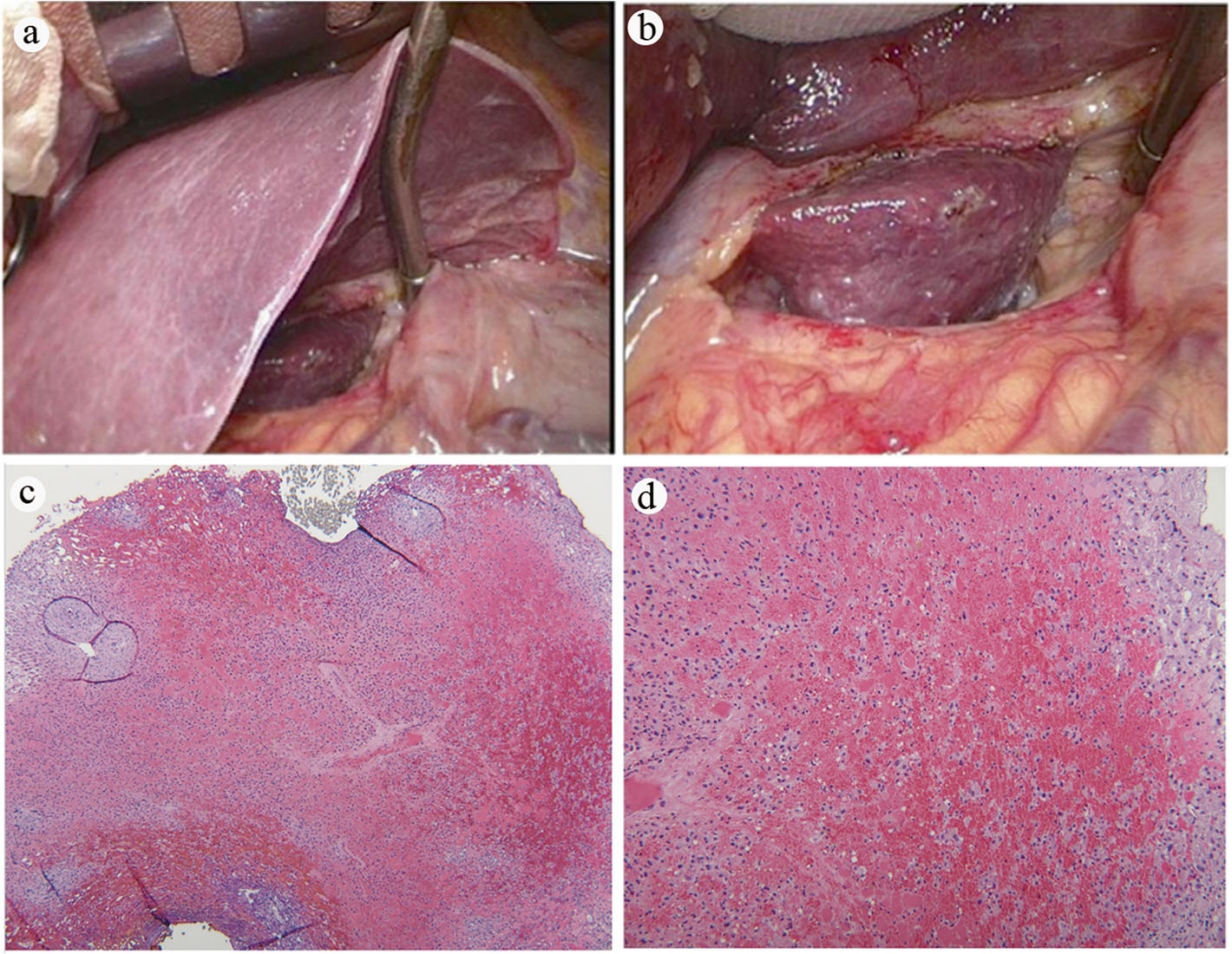
**a** The normal liver compared with the inflammatory caudate lobe. **b** Up-close view of the liver with dark red, soft, bloody infiltration over the caudate lobe. Pathology of the liver caudate lobe. **c** Low-power field shows no evidence of tumor metastasis. Original magnification × 20. **d** High-power field indicates congestion with attenuated hepatic cords and filled with erythrocytes. Original magnification × 40

The tumor markers, including α-fetoprotein (5.74 ng/ml), carcinoembryonic antigen (3.27 ng/ml), and squamous cell carcinoma–related antigen (0.3 ng/ml), disclosed nothing remarkable. A further imaging survey was performed to clarify the new lesion. Liver sonography (Fig. 4a) showed focally mild prominence in S1 of the liver with a slightly hypoechoic appearance, and CT of the liver revealed focally less enhancement in S1 of the liver (Fig. 4b). Additionally, magnetic resonance imaging (MRI) of the liver showed a low signal intensity on T1-weighted images, along with a high signal intensity on T2-weighted images, over the caudate lobe (Fig. 4c, d). The liver MRI in the formal report indicated the new lesion at the liver over S1 as being due to postradiation liver inflammation change rather than liver metastasis from esophageal cancer. Excluding the possibility of distant metastasis, a thoracoscopic esophagectomy with cervical esophagogastrostomy via the retrosternal route, along with a feeding jejunostomy, was performed smoothly 2 weeks later. A liver biopsy for both frozen section and pathology was also conducted during the surgery. The pathology report revealed esophageal cancer, specifically poorly differentiated squamous cell carcinoma, pT3N1M0. A valuable photograph was taken during surgery that revealed inflammation of the caudate lobe. The photograph also showed a manifested dark red color due to liver congestion containing a soft, smooth surface without adherence to the surrounding tissue (Fig. 5a, b), with no palpable hard nodules being seen in the lesion. There was no obvious tumor metastasis or enlarged lymph node discovered in the abdomen during the operation. The histology elucidated sinusoidal congestion with attenuated hepatic cords, which were filled with erythrocytes. However, there was no evidence of liver metastasis (Fig. 5c, d). The patient recovered uneventfully and was discharged with his oral intake performing smoothly, and a stable condition was observed during 12 months of outpatient department follow-up.

## Discussion

The hepatic parenchyma is radiosensitive and has traditionally limited the use of RT in treating liver tumors or other perihepatic tumors located in the upper abdomen [9]. Preoperative whole-body PET-CT is frequently used to evaluate RT response and exclude metastases. Radiation-induced liver injury (RILI) may be detected by PET-CT within 2 to 6 weeks after completion of RT as being a focal area of increased FDG uptake (> 50% over baseline) in the liver adjacent to the irradiated field with an associated decreased attenuation seen by CT [5]. Common sites of metastatic spread in esophageal cancer include the abdominal lymph nodes, liver, and lungs [10]. In one study, it was revealed that up to 35% of patients with esophageal cancer will develop liver metastases [11]. In addition to metastatic disease, the liver is also particularly prone to radiation-induced liver disease (RILD) during RT for distal esophageal cancer, owing to its close proximity to the left lobe, which is accordingly included in the standard radiation field [11].

When a patient displays FDG activity in the left or caudate liver lobes after CRT, a more detailed investigation should be performed in order to confirm or exclude distant metastases [12]. Our patient displayed no signs of tumor metastasis prior to neoadjuvant CRT. However, a new FDG uptake lesion was discovered during PET-CT tumor restaging. Theoretically, the liver lesion may have been due to the chemotherapy, RT, or a synergic effect [4, 6, 13]. In patients with esophageal cancer undergoing neoadjuvant therapy, three-dimensional conformal therapy or intensity-modulated RT is employed in order to increase the radiation dose to the primary tumor while limiting damage to the surrounding healthy tissue [14]. However, given that the anatomical location of the lateral segment of the left lobe of the liver is adjacent to the distal esophagus, radiation injury to the liver is difficult to avoid [5]. There is an additional risk of RILD due to the concurrent administration of hepatotoxic chemotherapy to radiation, as well as irradiating a patient who has already received hepatotoxic chemotherapy prior to radiation [15]. However, cisplatin rarely results in hepatic toxicity (steatosis and cholestasis) when prescribed in standard doses [16]. Cisplatin induced hepatitis often elevates both aspartate transaminase (AST) and alanine transaminase (ALT) levels; however in our patient, the AST and ALT levels both before and after neoadjuvant CRT were within the normal range. Cisplatin-induced peliosis hepatis should affect the diffused liver rather than focusing on the caudate lobe due to the RT. With regard to a possible synergetic effect between cisplatin treatment and RT, additional research is still required. Radiation tolerance of the liver is less in patients with a deranged liver function [15]. These patients are more susceptible to the development of RILD [15].

Reed and Cox were the first to describe the pathophysiology of RILD and suggested that retrograde congestion was the main culprit [17]. Although RILD typically occurs 4–8 weeks after the termination of RT, it has been reported to appear as early as 2 weeks or as late as 7 months after RT [15]. In the available literature, RILI is described in about 8% of patients at the time of restaging. The precise mechanisms of RILD development remain largely unknown [18]. Although knowledge of RILD pathogenesis has improved in recent years, the molecular pathogenic mechanisms of RILD remain unclear [18]. RILD pathogenesis includes complex and multicellular responses associated with vascular changes, increased collagen synthesis, and sequential activation of key growth factors and cytokines, such as tumor necrosis factor-α, transforming growth factor-β, and Hedgehog, which are important regulators in repair responses to liver damage [19]. Radiation injury to the liver most likely begins with damage to the endothelial cells of the central veins and sinusoids, which leads to sinusoidal congestion. In its more advanced stages, veno-occlusive disease is seen, resulting in backflow congestion and liver necrosis [20, 21].

There are two types of RILD: classic RILD and nonclassic RILD [18]. Patients experiencing classic RILD usually exhibit symptoms of fatigue, abdominal pain, increased abdominal girth, hepatomegaly, and anicteric ascites 1–3 months after liver RT [22]. The pathological hallmark of classic RILD is hepatic veno-occlusive disease, which is characterized by the complete obliteration of the central vein lumina by erythrocytes trapped in a network of reticulin and collagen fibers [17, 23]. Patients who develop nonclassic RILD have underlying chronic hepatic diseases, such as cirrhosis and viral hepatitis, and display more dysregulated hepatic functions with jaundice and/or remarkably elevated serum transaminases [18]. For example, patients with the hepatitis B virus are reportedly more vulnerable than noncarrier groups to developing RILD [24].

A similar report by DeLappe *et al.* described a 61-year-old man with esophageal cancer who experienced a new increase in FDG uptake over the left lobe of the liver after undergoing 50.4-Gy RT, with the patient’s CT-guided liver biopsy revealing no liver metastasis [25]. In Oregon, USA, 112 patients diagnosed with distal esophageal cancer were treated with neoadjuvant CRT, with 10 of them detected as having increased FDG uptake during PET-CT tumor restaging; however, 1 patient was later diagnosed with interval metastasis, and the others experienced RILI with an abnormal FDG uptake in the left caudate lobe from the liver [13]. Additionally, 26 patients were treated with neoadjuvant CRT for esophageal cancer in the MD Anderson Cancer Center, where 2 of them experienced an increased FDG uptake in the left lobe of the liver, whereas no FDG uptake occurred at the right lobe of the liver [5]. In a study by Francine *et al.* involving 205 patients who were undergoing neoadjuvant chemoradiation therapy, 6 of the patients showed an increased FDG uptake in the caudate or left liver lobe as seen on a PET-CT scan. None of the patients had any detected liver metastasis, as proved by biopsy, additional images, or continuous follow-up [12].

Characterizing RILD through noninvasive imaging has been challenging, and the techniques are still evolving [26]. CT findings within the irradiated portion of the liver after conventionally fractionated RT consist of a reversible, generally well-demarcated region of reduced enhancement when compared with the corresponding liver, possibly representing an increased water or fat content in the irradiated liver [27–29]. Increased enhancement can also be seen in the irradiated liver when compared with the adjacent normal liver because of an increased arterial flow or a delayed contrast clearance from radiation-induced veno-occlusive disease [30]. RILD may present itself as demarcated areas of hypo- or hyperattenuation in a nonanatomic distribution [31]. The appearance of radiation injury on CT scans is quite characteristic and generally shows sharp, straight margins that correspond to the portals used [5]. Metastatic lesions, on the other hand, are generally more masslike and rounded in contour on CT scans [5]. Magnetic resonance imaging shows changes after liver irradiation [26]. Decreased signal intensity on T1-weighted images, increased signal intensity on T2-weighted images, and an increased signal intensity on proton spectroscopic imaging of irradiated liver lobes suggests that the irradiated liver has increased water content [28]. MRI produces high-resolution images with good soft tissue contrast, which is ideal for distinguishing organs from surrounding tissue [32]. Various clinical studies have investigated the use of MRI to detect and monitor radiation-induced damage to the liver [33], myocardium [34, 35], and bone marrow [36, 37]. New foci of hepatic FDG avidity that develops during neoadjuvant chemoradiation of esophageal cancer are usually due to RILD. Increased FDG avidity in RILD results from inflammation caused by radiation, with an increased FDG uptake by active leukocytes [25]. This reflects the low likelihood of metastases developing during neoadjuvant CRT [13]. The location of the new foci of FDG avidity may be important. All the foci due to RILD developed in the left and caudate lobes, within the presumed radiation field [13]. The involved organ will be related to chemoradiation-induced injuries, though the exact frequency with consensus is not made clear in the literature, but vulnerable organs include the lung, liver, heart, spinal cord, kidney, and bowel, as well as others. The dose of radiation causing RILD has ranged from a pure dose to 70 cGy, combining chemotherapy with 30 cGy, or in underlying liver disease with a de-escalating dose. Currently, no special report is available to compare the maximum standardized uptake value (SUVmax) in PET-CT between radiation injury and metastatic lesions, but some studies have revealed an SUVmax of RILD range at about 4–9/hour, but metastatic lesions can sometimes be higher than 10/hour; if SUVmax is more than 10/hour, it must be due to metastasis.

## Conclusions

New foci from increased FDG avidity are commonly seen in the caudate and left hepatic lobes of the liver during neoadjuvant chemoradiation of distal esophageal cancer, and these findings can be RILD rather than metastatic disease [13]. Awareness of the pitfalls of a high FDG uptake in RILI is crucial in order to avoid misinterpretation and overstaging. Except for the location of FDG uptake, the shape of the lesion, and a SUVmax value (> 10/hour), a convincing liver MRI or even a liver biopsy can provide accurate information to help distinguish RILI from liver metastasis.

## Data Availability

Data sharing is not applicable to this article, because no datasets were generated or analyzed during the current study.

## Abbreviations

ALT: Alanine transaminase
AST: Aspartate transaminase
CRT: Chemoradiotherapy
CT: Computed tomography
FDG: ^18^F-fluorodeoxyglucose
MRI: Magnetic resonance imaging
PET-CT: Positron emission tomography with computed tomography
RILD: Radiation-induced liver disease
RILI: Radiation-induced liver injury
RT: Radiation therapy
SUVmax: Maximum standardized uptake value
